# The economic benefits of reducing physical inactivity: an Australian example

**DOI:** 10.1186/1479-5868-8-99

**Published:** 2011-09-24

**Authors:** Dominique A Cadilhac, Toby B Cumming, Lauren Sheppard, Dora C Pearce, Rob Carter, Anne Magnus

**Affiliations:** 1 Stroke and Ageing Research Centre, Southern Clinical School, Monash University, Clayton 3168, Vic, Australia; 2 National Stroke Research Institute, Heidelberg Heights 3081, Vic, Australia; 3 Deakin Health Economics, Deakin University, Burwood Australia; 4 The University of Melbourne 3010, Australia

## Abstract

**Background:**

Physical inactivity has major impacts on health and productivity. Our aim was to estimate the health and economic benefits of reducing the prevalence of physical inactivity in the 2008 Australian adult population. The economic benefits were estimated as 'opportunity cost savings', which represent resources utilized in the treatment of preventable disease that are potentially available for re-direction to another purpose from fewer incident cases of disease occurring in communities.

**Methods:**

Simulation models were developed to show the effect of a 10% feasible, reduction target for physical inactivity from current Australian levels (70%). Lifetime cohort health benefits were estimated as fewer incident cases of inactivity-related diseases; deaths; and Disability Adjusted Life Years (DALYs) by age and sex. Opportunity costs were estimated as health sector cost impacts, as well as paid and unpaid production gains and leisure impacts from fewer disease events associated with reduced physical inactivity. Workforce production gains were estimated by comparing surveyed participation and absenteeism rates of physically active and inactive adults, and valued using the friction cost approach. The impact of an improvement in health status on unpaid household production and leisure time were modeled from time use survey data, as applied to the exposed and non-exposed population subgroups and valued by suitable proxy. Potential costs associated with interventions to increase physical activity were not included. Multivariable uncertainty analyses and univariate sensitivity analyses were undertaken to provide information on the strength of the conclusions.

**Results:**

A 10% reduction in physical inactivity would result in 6,000 fewer incident cases of disease, 2,000 fewer deaths, 25,000 fewer DALYs and provide gains in working days (114,000), days of home-based production (180,000) while conferring a AUD96 million reduction in health sector costs. Lifetime potential opportunity cost savings in workforce production (AUD12 million), home-based production (AUD71 million) and leisure-based production (AUD79 million) was estimated (total AUD162 million 95% uncertainty interval AUD136 million, AUD196 million).

**Conclusions:**

Opportunity cost savings and health benefits conservatively estimated from a reduction in population-level physical inactivity may be substantial. The largest savings will benefit individuals in the form of unpaid production and leisure gains, followed by the health sector, business and government.

## Background

Physical activity, which is increasingly being engineered out of our working and social lives, is important to maintaining health. Physical activity enhances muscle strength, aerobic capacity and psychological well-being, while moderating health risk factors such as obesity, high cholesterol and hypertension [1]. Physical activity levels equivalent to 2.5 hours per week of moderate-intensity activity (i.e. an effort equivalent to brisk walking, or approximately 4000 kJ/week) are considered important targets to achieve health benefits [2]. Evidence suggests that walking for half an hour a day, five days a week, may increase life expectancy by 1.5 to 3 years depending on the intensity [3]. The time lag between increasing physical activity and observing health benefits is relatively short [4,5]. However, many people do not participate in regular physical activity. The two main barriers appear to be time limitations and dissatisfaction, since many do not enjoy exercise [6]. In Australia, there is evidence that 70% of adults are either sedentary or have a low activity level [7]. Evidence from three national health surveys conducted between 1995 and 2005 suggest that the proportions of Australians reporting sedentary or low exercise levels have not changed markedly over the last ten years [7,8].

A sedentary lifestyle has been associated with a greater risk of all-cause mortality [9]. Inactivity is also associated with increased risk of cardiovascular disease [10], ischemic stroke [11], non-insulin-dependent (type 2) diabetes [12], colon cancer [13], osteoporosis [14], hip fracture following falls [15] and depression [16]. Nearly 7% of Australia's health burden has been attributed to physical inactivity, with the greatest contributors being ischemic heart disease (51%), type 2 diabetes (20%) and stroke (14%) [2]. Therefore, encouraging increased physical activity levels is important. A range of interventions are effective for reducing inactivity, including those that provide professional guidance and on-going support [17], targeted information, behavioral and social interventions (e.g. community based social support programs), and environmental and policy interventions [18,19].

Few authors have quantified the economic costs of physical inactivity and the value of increasing participation in physical activity to levels that produce health benefits. In studies from Canada, Switzerland, the United Kingdom (UK) and United States (US), annual direct healthcare costs attributable to physical inactivity ranged from 1.5% to 3% of total direct health costs [20]. Regular physical activity can improve musculoskeletal and cardiovascular health and enhance mental well being, which in a population improves general health and productivity. Governments can benefit through future savings in avoidable health care expenditure, increased income taxation and fewer welfare payments. Businesses benefit from reduced absenteeism and lower recruitment and training costs associated with replacing staff, and individuals benefit from more income and increased quality of life.

The aim of this study was to quantify the health and economic benefits that could be achieved following a feasible reduction (as opposed to a complete elimination) of physical inactivity in the Australian adult population. This study was part of a larger study funded by the Victorian health promotion foundation (VicHealth) whereby the benefits of feasible reductions in the prevalence of alcohol, physical inactivity, high body mass index, tobacco smoking, inadequate consumption of fruit and vegetables, and intimate partner violence were estimated [21].

## Methods

To estimate the health and economic benefits to society, the impact of an absolute reduction in the prevalence of physical inactivity levels for the 2008 Australian population was measured as reduced incident cases of preventable physical inactivity-related diseases, deaths and disability adjusted life years (DALYs). Relevant diseases affected included cardiovascular disease, cancers, fractures and depression.

The initial step was to agree on a feasible target reduction in the prevalence of physical inactivity. The feasible reduction targets selected for physical inactivity in Australia were based on expert consensus via discussions with the study-specific Advisory Committee and consultation with health promotion experts coupled with a review of the broader intervention-based literature. The evidence from the literature indicated that health benefits should accrue when the prevalence of physical inactivity is reduced by 5% to 10%. In the systematic review of Kahn et al. [18], there were 5 studies that used community-wide intervention campaigns and reported change in the percentage of people being active. The median net increase was 4.2%, with one study reporting an increase of 9.4%. In 2000, the US set the objective of increasing the percentage of people doing at least 30 minutes of moderate physical activity regularly from 15% to 30% over 10 years [22]. Individually targeted interventions have yielded greater increases in activity, with differences in the region of 20-30 percentage points between intervention and control groups in the proportion of people walking for at least 20 minutes per day at least 3 times per week [23,24]. Stephenson et al [25] nominated 5% and 10% point shifts in prevalence as part of their sensitivity analyses of the benefits of increased physical activity in an Australian cost-of-illness study. Similarly, Katzmarcyk et al aimed for a reduction of 10% in inactivity levels in Canada [26]. Another option we considered was to make a comparison with another country using the concept of an 'Arcadian ideal' [27]. However, this approach was judged less robust since: a) we could not identify a country that was demographically and culturally comparable to Australia and where the prevalence of physical inactivity was lower; and b) the variations in the definition of 'physical inactivity' meant we could not be certain that such a comparison was valid. In Australia, the recommended level of physical activity is defined as 3 sessions of at least 20 minutes vigorous exercise or 5 sessions of at least 30 minutes moderate exercise per week [2]. Thus a 10% reduction in physical inactivity was selected as an ideal feasible target, and a progressive target of 5% were both modeled.

The comparator groups analyzed were the exposed Australian population of inactive people (defined as sedentary or low activity category that did not meet recommended activity levels), and the non-exposed active population (defined as moderate to high activity that met or exceeded recommended activity levels).

The net difference in mortality, incident morbidity and consequent health sector costs and the impact on paid and unpaid production and leisure between the current prevalence of physical inactivity and the two target prevalence levels for the 2008 Australian adult population was then estimated with population-based simulation models developed in Excel (Microsoft Corporation, 2003). Cost data from other years were adjusted to 2008 by applying health price inflators [28]. A 3% discount rate for lifetime benefits was applied [29], and varied in sensitivity analyses using 0%, 5% and 7% (data not reported but available from the authors).

### Simulation models and data analyses

The Workforce Production Gains model developed by Magnus et al [30] was adapted in the current study to estimate the production gains/losses and taxation effects in the Australian economy if a target reduction in physical inactivity prevalence were achieved. The model includes simulation of a theoretical cohort of Australians (ages 15-65 years) during their working years until retirement age. In this model the working lifetime income earned and taxation paid is calculated taking into account known participation rates and absenteeism rates by age and sex of the exposed and the non-exposed sub populations. The production gains or losses arise from changes in income earned and taxation paid that result from the reduction in deaths and incident cases of disease and disability, associated with the reduction in prevalence of physical inactivity in the adult population. Two methodological techniques were used to value the production gains or losses. The Friction Cost Approach (FCA) assumes individuals who die or leave the work force due to disability (for example, following a stroke) will be replaced after a specified period resulting in shorter term production losses to society. As a sensitivity analysis, the second technique adopted to value production gains or losses was the Human Capital Approach (HCA) which counts all future income up to age 65, as lost from an individual who leaves the workforce due to death or disability. There remains debate in the economic literature about which method is preferable [31,32] since they give such divergent results. For the purposes of the current study, the FCA had a stronger logical connection to the actual likely cost impact on industry and was considered to provide a more realistic economic estimate. Three months was used as the friction period [31,33] and was varied to 6 months in sensitivity analyses.

The Household Production and Leisure Time model was developed to estimate the net difference in the economic value of hours of lost leisure and household production associated with diseases attributable to physical inactivity. The model incorporated surveyed time allocations of both working and non-working adults by gender and age. Household production was defined as the hours spent performing non-paid household duties such as cooking, shopping, cleaning, child care and maintenance. These were valued at 'replacement cost', whereby the duties unable to be performed due to illness were purchased commercially. Unit prices for household production were based on the average 2008 wage rates for domestic services and child care. Leisure time comprised social and community interaction, together with recreation and leisure activities only. Leisure time was valued using the 'opportunity cost method', applying one third of the average 2008 weekly earnings for men and women [34]. The National Health Survey (NHS) provided self-reported days out of role for the exposed (inactive) and non-exposed (active) Australian populations. It was assumed on these days that the household duties and leisure time normally performed, would not be performed, involving an economic loss of leisure time and the need to replace the household activities commercially. Following the reduction in the prevalence of physically inactive persons in the Australian population there was a change in the number of days of household and leisure activities lost to ill health. The net difference in the value of the days of household production and leisure time between the current prevalence and targeted physically inactive and active prevalence was counted as the economic gain.

### Health sector cost estimation

To estimate changes in health sector costs, the attributable portion of total health sector costs to diseases associated with physical inactivity were estimated using Population Attributable Fractions (PAF) [2]. A PAF is the proportion by which the incidence of a disease in a population could be reduced if the risk factor or exposure was to reach a 'theoretical minimum' - the lowest possible level of risk in a population [2]. Calculation of a PAF is informed by epidemiologic studies where relative risk estimates for disease have been reliably determined for people exposed and not exposed to single risk factors. In the current study, PAFs for diseases attributable to physical inactivity were taken for males and females by age group from the 2003 Australian Burden of Disease study [2]. The modeling of lifetime health expenditure costs from these data was not attempted. Rather, a conservative approach was taken, where only the annual health sector costs of treating incident cases of disease attributable to physical inactivity were assumed to approximate the health sector cost savings of a reduction in the prevalence of physical inactivity for our reference (2008) population.

### Data sources

The most up-to-date Australian data sources were used. The current estimates for the prevalence of physical inactivity (overall 70%) by age and gender were obtained from the 2004/5 NHS [7] confidentialized dataset with the approval of the Australian Statistician, Australian Bureau of Statistics (ABS) [7,35]. Respondents self reported how much exercise they had undertaken in the two weeks prior to the survey and categorized their exercise according to intensity.

Demographic data, employment status, and health-related actions of physically inactive and active adults were also obtained from the 2004/5 NHS dataset (Table 1). PAFs, health status estimates including incident cases of physical inactivity-related disease, deaths and DALYs were obtained using the 2003 Australian Burden of Disease data files [2] that were made available for this study. The 2000-01 Disease Costs and Impact Study Excel files [36], which adopted the Burden of Disease classification system, were used to estimate the change in health sector costs from diseases associated with physical inactivity. Household production and leisure time were derived from the 2006 ABS Time Use Survey as described earlier [37]. Current average wages were sourced from the ABS and published government pay scale summaries [38,39].

**Table 1:** Demographics and days of reduced activity due to ill health by age, gender and work force status for the 2008 adult Australian population

	Male		Female	
	**Physically inactive**	**Physically active**	**Physically inactive**	**Physically active**

**Age summary**				
Age 15-64 y N (95% CI)	4,332,994 (4,229,842 - 4,436,146)	2,322,617 (2,220,059 - 2,425,175)	4,798,508 (4,704,081 - 4,892,935)	1,864,120 (1,769,404 - 1,958,836)
Age 65+ y N (95% CI)	775,817 (746,962 - 804,671)	342,985 (313,395 - 372,575)	1,059,103 (1,028,507 - 1,089,698)	260,571 (230,005 - 291,136)
Mean age (15+ years) (95% CI)	44.9 (44.6 - 45.3)	40.6 (40.1 - 41.2)	45.3 (45.0 - 45.6)	42.5 (41.6 - 43.3)
In Labour Force (15+ years)*				
% (95% CI)	75% (73% - 76%)	75% (73% - 77%)	57% (56% - 58%)	65% (63% - 67%)
Mean days off work (95% CI)	0.32 (0.26 - 0.38)	0.26 (0.18 - 0.34)	0.31 (0.26 - 0.35)	0.23 (0.16 - 0.29)
**Not in Labour Force**				
% (95% CI)	26% (24% - 27%)	25% (23% - 27%)	43% (42% - 44%)	35% (33% - 37%)
Mean days of reduced activity: 15-64 y (95% CI)	1.93 (1.60 - 2.26)	0.88 (0.54 - 1.21)	1.45 (1.26 - 1.65)	0.84 (0.62 - 1.05)
**Aged 65+ years**				
% (95% CI)	15.2% (14.6% - 15.8%)	12.9% (11.8% - 14.0%)	18.1% (17.5% - 18.7%)	12.3% (10.8% - 13.8%)
Mean days of reduced activity (95% CI)	1.56 (1.25 - 1.87)	0.43 (0.22 - 0.65)	1.75 (1.53 - 1.96)	0.73 (0.50 - 0.96)

Source: National Health Survey 2004-05 (ABS, 2006); CI: Confidence Interval; N: Number. Mean days measured over a two week period. *includes unemployed seeking work and 65+ years

### Uncertainty analyses

Multivariable probabilistic uncertainty analyses were undertaken using @RISK software version 4.5 for Excel (Palisade Corporation, 2005). Input variables were modeled as known distributions rather than single values where uncertainty existed (e.g. each surveyed parameter and life-years remaining). Uncertainty in wages, participation rates and absenteeism were captured in the reported survey standard errors [7,38,39]. Monte Carlo sampling with minimum 4,000 simulations were used to estimate a mean and 95% uncertainty interval for the outcome parameters.

Comprehensive details on our methods are provided in the full technical report [21] available at http://www.vichealth.vic.gov.au/~/media/ResourceCentre/PublicationsandResources/Knowledge/Research%20Report_FINAL_July09.ashx and are also reported in the publication on tobacco smoking and the combined analysis of the six risk factors [40,41].

## Results

Table 1 presents the demographic data and days of reduced activity for physically active and physically inactive persons by age, gender and workforce status. Physically inactive females participated less in the workforce than physically active females. Among the physically inactive females in the workforce, more took days off work compared with physically active females. In addition, the physically inactive females not in the workforce had more days of reduced activity when compared to physically active females not in the workforce. Physically inactive males, who were not in the workforce, had more days of reduced activity compared to physically active males.

If the prevalence of physical inactivity in the adult Australian population was reduced by 10%, the estimated 45,000 annual new cases of physical inactivity-related disease could be reduced by 6,000 (13%); the 13,000 annual deaths attributed to physical inactivity could be reduced by 2,000 deaths (15%); and the 174,000 DALYs lost from physical activity could be reduced by about 25,000 (14%) (Table 2). Half of these benefits would be achieved if the progressive target (5% reduction) in the prevalence of physical inactivity was met.

**Table 2:** Health status and production effects from reducing the prevalence of physical inactivity

**Benefit**	**Feasible reduction target^a^**		
	
		**95% Confidence Interval**	
	
	**Mean ('000s)**	**Lower Limit ('000s)**	**Upper Limit ('000s)**
	
DALYs	25	n/a	n/a
Incidence of disease	6	n/a	n/a
Mortality	2	n/a	n/a
***Lifetime***			
Leisure (days)	316	300	331
Absenteeism (days)	114	n/a	n/a
Days out of home based production role (days)	180	155	206
Early retirement (persons)	0.02	n/a	n/a
	
	**Progressive target reduction**		
	
DALYs	12	n/a	n/a
Incidence of disease	3	n/a	n/a
Mortality	1	n/a	n/a
***Lifetime***			
Leisure (days)	158	150	166
Absenteeism (days)	57	n/a	n/a
Days out of home based production role (days)	90	77	103
Early retirement (persons)	0.01	n/a	n/a

^a^10% net reduction in prevalence. Disability Adjusted Life Years (DALYs), incidence of disease and mortality calculated for all age groups. Leisure and home based production calculated for persons aged 15+ years. Absenteeism and early retirement calculated for persons aged 15-64 years. All estimates uncorrected for joint effects.

**Figure 1:**
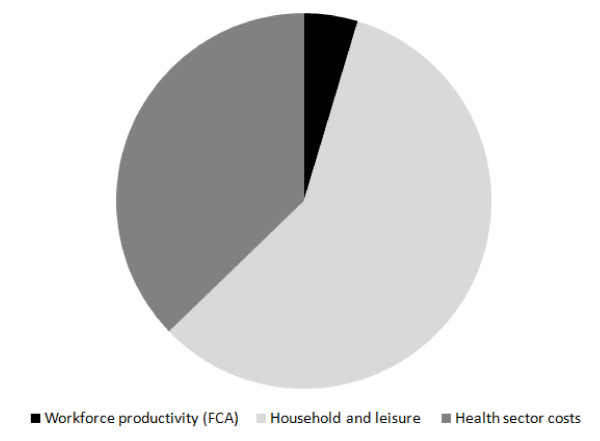
**Proportion of opportunity cost savings from reductions in physical inactivity by economic category**. FCA: Friction cost approach

The estimated benefits from reduced physical inactivity resulted in potential opportunity cost savings of AUD96 million to the health sector (0.19% of total annual health sector costs and 14% of attributable annual health sector costs to this risk factor), AUD12 million in workforce production (FCA), AUD71 million in home based production and AUD79 million in leisure based production (Table 3). This represents approximately 14% of the total production cost losses attributable to this risk factor (AUD1,135 million). The largest component of these total potential opportunity cost savings would occur in household production and leisure, followed by the health sector and workforce (Figure 1). AUD in 2008 can be converted to US dollars, using the purchasing power parity of AUD1.48 [42].

**Table 3:** Economic outcomes from reducing the prevalence of physical inactivity

**Economic outcomes**	**Feasible reduction target^a^**		
	
		**95% Confidence Interval**	
	
	**Mean** **(AUD million)**	**Lower Limit** **(AUD million)**	**Upper Limit** **(AUD million)**
	
Health sector costs	96	n/a	n/a
Production Costs FCA	12	7	18
Recruitment and training costs	5	n/a	n/a
Taxation effects FCA^b^	2	1	4
Leisure based production	79	60	103
Home based production	71	61	82
Total production FCA^c^	162	136	192
*Sensitivity analysis*			
Production Costs HCA	138	114	161
Taxation effects HCA^b^	12	10	15
Total production HCA^c^	288	253	326
			
	
	**Progressive target reduction**		
	
Health sector costs	48	n/a	n/a
Production Costs FCA	6	3	9
Recruitment and training costs	2	n/a	n/a
Taxation effects FCA^b^	1	0.26	2
Leisure based production	40	30	51
Home based production	36	30	41
Total production FCA^c^	81	68	96
*Sensitivity analysis*			
Production Costs HCA	69	58	81
Taxation effects HCA^b^	6	5	8
Total production HCA^c^	145	127	164

^a^These are not estimates of immediately realizable cash savings ^b^Taxation is treated as a transfer payment and should not be added to production effects. ^c^Total production is the sum of workforce production costs, household- and leisure-based production. All estimates uncorrected for joint effects of the presence of multiple risk factors in individuals. HCA: Human Capital Approach; FCA Friction Cost Approach (preferred conservative estimate). Health sector, leisure and home based production based on persons 15+ years. Production, recruitment and training and taxation effects based on persons 15-64 years.

## Discussion

The primary finding of this study is that a feasible reduction in prevalence of physical inactivity can lead to total potential opportunity cost savings of AUD258 million, with 37% of the savings arising in the health sector. The largest savings would benefit individuals, followed by the health sector, business and government. These savings would be much larger if all physical inactivity was eliminated (AUD672 million in health sector, AUD1,135 million [FCA] in production and leisure), but our aim was to estimate savings that were realistic and relevant to the setting of future public health campaigns and disease prevention strategies.

A novel and important aspect of the present study was the inclusion of benefits for workforce, household production and leisure time associated with reduced physical inactivity. The choice of appropriate methods for quantifying and valuing household production and leisure time continue to be debated [43]. Nevertheless, capturing household and leisure activities is increasingly regarded by health promotion agencies as essential to appropriate population-level economic modeling. Indeed, this element was identified as the largest component of the total opportunity cost savings to be made through increased physical activity. This occurs because the avoidable diseases associated with physical inactivity are largely diseases occurring in older age groups. Older individuals no longer in the workforce have potentially the most to gain from increasing their levels of physical activity, including quality of life. This is particularly relevant in ageing populations (most of the developed nations) where rises in future health sector costs are driven by the increased demand of an ageing population. In addition, inclusion of these estimates is also important to not underestimate the health and economic impacts of increasing physical activity in a community since there is a gender bias to workforce participation and obvious differences among females who are and are not in the workforce, and who are physically active and inactive.

It is difficult to compare these estimates of potential savings with earlier literature. The only previous Australian study to identify health sector costs associated with physical inactivity estimated them at AUD377 million [25]. The largest costs arose from preventing coronary heart disease (AUD161 million) and stroke (AUD101 million). In contrast, the current study was used to estimated the *annual *health sector cost attributable to incident cases of physical inactivity at AUD672 million (representing 1.3% of total annual health sector costs). While the results are not directly comparable given fundamental methodological differences, both studies provide a valuable contribution to the literature regarding the potential economic benefits of reducing physical inactivity in Australia.

Other strengths of this work were the conservative approach taken and the use of best available data. It was assumed that only *incident *cases of disease attributable to physical inactivity for the 2008 population would be reduced. Thus, future reductions in disease risk among those already ill (e.g. benefits gained from reducing inactivity in people with existing cardiovascular disease) were not incorporated in these analyses. Furthermore, the impact on health sector costs for reduced mortality is not easily modeled, since death-related costs occur during a lifetime, rather than at the point of death. The more conservative FCA estimates of workforce production gains over the HCA were preferred by the investigators of this present study, and represented less than 10% of the HCA estimates.

The reliance on self-reported cross-sectional data is a limitation, since such data are less reliable than objective measurement data, because people can exaggerate, fail to remember, or misunderstand questions. The direction of reporting bias is not always clear. In addition, other concurrent risk factors and socioeconomic status, not controlled for in this analysis, could be a source of overestimation. It is also possible that people increase their level of activity following the onset of an illness (e.g. diabetes, cardiovascular disease). Assuming causality in the absence of rigorous longitudinal data means that the results must be regarded as broadly indicative of what might be achieved. A further limitation is that forecasted gains will occur over time. A quantitative assessment of when opportunity cost savings and health status benefits would be achieved was not undertaken. This approach is rare because of the subsequent additional levels of uncertainty, making estimates less reliable. However, there is some evidence that the time lag between increased physical activity and observed benefits is relatively short. Blair demonstrated that increasing activity reduced all cause mortality within two years, which was half the time required to observe benefits from smoking cessation [4].

The selection of targets for risk factor prevalence reduction is an important policy decision. It is possible that a 10% reduction in physical inactivity prevalence is an overly ambitious goal in the current Australian climate of increasing levels of obesity, with modern technology eliminating the need for many physical pursuits. However, this bold target could be justified as feasible given the high prevalence of inactivity.

Lastly, opportunity cost savings need to be carefully interpreted. These savings will only be achieved by the adoption of effective interventions that will invariably have implementation and time costs attached to them. Including intervention costs and effects was beyond the scope of this study and it was assumed that acceptable effective interventions exist to achieve the target reductions in physical inactivity. Opportunity cost savings are not estimates of immediately realizable financial savings; they are estimates of resources consumed in current practice that could be made available for other purposes, such as investing in public health programs. Future, well-designed epidemiological and clinical research studies are needed to provide better evidence to underpin decision analytic modeling for health promotion, and for prioritizing specific interventions to achieve reductions in inactivity.

Investment in disease prevention and health promotion in Australia is dwarfed by avoidable spending on disease treatment. The findings of this project contribute important new knowledge about the major impact of physical inactivity on the productivity of individuals in both the paid and unpaid sectors, as well as health sector expenditure. The findings from this study reinforce the argument that greater investment in risk factor reduction strategies is required and economically justified, particularly in ageing populations.

## Competing interests

The authors declare that they have no competing interests.

## Authors' contributions

AM, DC and RC conceptualized the project. AM, DC, TC and LS conducted literature reviews. AM and DC developed the economic models. DP performed the data analyses. DC and TC wrote the initial draft of the paper. All authors reviewed and contributed to drafts of the paper. All authors have read and approved the final manuscript.

## Authors' Information

DC: Head Translational Public Health Unit, Stroke and Ageing Research Centre, Monash University, Victoria. Head: Public Health. National Stroke Research Institute, Heidelberg Heights, Victoria, Australia. Honorary Research Fellow: Department of Medicine, The University of Melbourne, Australia and Deakin Health Economics, Strategic Research Centre Population Health, Faculty of Health, Deakin University, Burwood Deakin University and The University of Melbourne, Parkville, 3010.

TC: Post-doctoral Research Fellow. National Stroke Research Institute, Heidelberg 3084, Victoria, Australia

LS: Research Fellow. Deakin Health Economics, Strategic Research Centre Population Health, Faculty of Health, Deakin University, Burwood, 3125, Victoria, Australia

DP: Research Fellow, Melbourne School of Population Health, The University of Melbourne, 3010, Parkville, Victoria, Australia.

RC: Founding Chair, Deakin Health Economics, Strategic Research Centre Population Health, Faculty of Health, Deakin University, Burwood, 3125, Victoria, Australia

AM: Senior Research Fellow, Deakin Health Economics, Strategic Research Centre Population Health, Faculty of Health, Deakin University, Burwood, 3125, Victoria, Australia

## References

[B1] Taylor AH Cable NT Faulkner G Hillsdon M Narici M Van Der Bij AK Physical activity and older adults: a review of health benefits and the effectiveness of interventions Journal of Sports Sciences 2004 22 8 703 725 10.1080/02640410410001712421 15370483

[B2] Begg S Vos T Barker B Stevenson C Stanley L Lopez AD AIHW C The burden of disease and injury in Australia 2003 Volume PHE 82 2007

[B3] Franco OH de Laet C Peeters A Jonker J Mackenbach J Nusselder W Effects of physical activity on life expectancy with cardiovascular disease Arch Intern Med 2005 165 20 2355 2360 10.1001/archinte.165.20.2355 16287764

[B4] Blair SN Kohl HW Barlow CE Paffenbarger RS Jr Gibbons LW Macera CA Changes in physical fitness and all-cause mortality. A prospective study of healthy and unhealthy men.[see comment] JAMA 1995 273 14 1093 1098 10.1001/jama.273.14.1093 7707596

[B5] Paffenbarger RS Jr Hyde RT Wing AL Lee IM Jung DL Kampert JB The association of changes in physical-activity level and other lifestyle characteristics with mortality among men.[see comment] New England Journal of Medicine 1993 328 8 538 545 10.1056/NEJM199302253280804 8426621

[B6] Meltzer DO Jena AB The economics of intense exercise J Health Econ 2010 29 3 347 352 10.1016/j.jhealeco.2010.03.005 20371127 PMC2864796

[B7] Australian Bureau of Statistics Australian Bureau of Statistics. Canberra National Health Survey 2004-05 Volume Cat No 4364.0 2006

[B8] Australian Bureau of Statistics Australian Bureau of Statistics. Canberra National Health Survey 1995: Summary of Results Australia Volume Cat No 4364.0 1997 75

[B9] Blair SN Kohl HW Paffenbarger RS Jr Clark DG Cooper KH Gibbons LW Physical fitness and all-cause mortality. A prospective study of healthy men and women JAMA 1989 262 17 2395 2401 10.1001/jama.262.17.2395 2795824

[B10] Wannamethee SG Shaper AG Physical activity in the prevention of cardiovascular disease: an epidemiological perspective Sports Medicine 2001 31 2 101 114 10.2165/00007256-200131020-00003 11227978

[B11] Gorelick PB Sacco RL Smith DB Alberts M Mustone-Alexander L Rader D Ross JL Raps E Ozer MN Brass LM Prevention of a first stroke: a review of guidelines and a multidisciplinary consensus statement from the National Stroke Association JAMA 1999 281 12 1112 1120 10.1001/jama.281.12.1112 10188663

[B12] Helmrich SP Ragland DR Leung RW Paffenbarger RS Jr Physical activity and reduced occurrence of non-insulin-dependent diabetes mellitus New England Journal of Medicine 1991 325 3 147 152 10.1056/NEJM199107183250302 2052059

[B13] Giovannucci E Ascherio A Rimm EB Colditz GA Stampfer MJ Willett WC Physical activity, obesity, and risk for colon cancer and adenoma in men Annals of Internal Medicine 1995 122 5 327 334 7847643 10.7326/0003-4819-122-5-199503010-00002

[B14] Kohrt WM Snead DB Slatopolsky E Birge SJ Jr Additive effects of weight-bearing exercise and estrogen on bone mineral density in older women Journal of Bone & Mineral Research 1995 10 9 1303 1311 7502701 10.1002/jbmr.5650100906

[B15] Jaglal SB Kreiger N Darlington G Past and recent physical activity and risk of hip fracture American Journal of Epidemiology 1993 138 2 107 118 8342529 10.1093/oxfordjournals.aje.a116833

[B16] Camacho TC Roberts RE Lazarus NB Kaplan GA Cohen RD Physical activity and depression: evidence from the Alameda County Study American Journal of Epidemiology 1991 134 2 220 231 1862805 10.1093/oxfordjournals.aje.a116074

[B17] Foster C Hillsdon M Thorogood M Interventions for promoting physical activity Cochrane Database of Systematic Reviews 2005 1 CD003180 10.1002/14651858.CD003180.pub2 PMC4164373 15674903

[B18] Kahn EB Ramsey LT Brownson RC Heath GW Howze EH Powell KE Stone EJ Rajab MW Corso P The effectiveness of interventions to increase physical activity. A systematic review American Journal of Preventive Medicine 2002 22 4 Suppl 73 107 11985936 10.1016/s0749-3797(02)00434-8

[B19] Graves N Barnett AG Halton KA Veerman JL Winkler E Owen N Reeves MM Marshall A Eakin E Cost-effectiveness of a telephone-delivered intervention for physical activity and diet PLoS One 2009 4 9 e7135 10.1371/journal.pone.0007135 19779611 PMC2744997

[B20] Oldridge NB Economic burden of physical inactivity: healthcare costs associated with cardiovascular disease European Journal of Cardiovascular Prevention & Rehabilitation 2008 15 2 130 139 10.1097/HJR.0b013e3282f19d42 18391637

[B21] Cadilhac D Cumming T Sheppard L Pearce D Carter R The economic benefits of reducing disease risk factors 2009 Melbourne: Deakin Health Economics Group, Deakin University and National Stroke Research Institute

[B22] United States Department of Health and Human Services Healthy People 2010: Conference edition 2000 Washington, DC: Government Printing Office

[B23] Dubbert PM Cooper KM Kirchner KA Meydrech EF Bilbrew D Effects of nurse counseling on walking for exercise in elderly primary care patients J Gerontol A Biol Sci Med Sci 2002 57 11 M733 740 10.1093/gerona/57.11.M733 12403802

[B24] Lombard DN Lombard TN Winett RA Walking to meet health guidelines: the effect of prompting frequency and prompt structure Health Psychol 1995 14 2 164 170 7789352 10.1037//0278-6133.14.2.164

[B25] Stephenson J Bauman A Armstrong T Smith B Bellew B Department of Health and Aged Care & Australian Sports Commission The costs of illness attributable to physical inactivity in Australia: A preliminary study 2000

[B26] Katzmarzyk PT Gledhill N Shephard RJ The economic burden of physical inactivity in Canada.[see comment] CMAJ Canadian Medical Association Journal 2000 163 11 1435 1440 PMC80410 11192648

[B27] Armstrong B McNeil JJ, King R, Jennings G, Powles J Morbidity and mortality in Australia: how much is preventable? Text book of preventive medicine 1990 Melbourne: Edwin Arnold 340

[B28] AIHW Australian Institute of Health and Welfare. Canberra Public health expenditure in Australia 2006-07 Volume AIHW Cat no HWE 41 2008

[B29] Gold MR Siegel JE Russell LB Weinstein MC Cost-effectiveness in health and medicine 1996 New York: Oxford University Press

[B30] Magnus A Mihalopoulos C Carter R Evaluation of preventive health interventions: Impact on production gains 2008 Melbourne: Deakin Health Economics Unit

[B31] Koopmanschap MA Rutten FF A practical guide for calculating indirect costs of disease Pharmacoeconomics 1996 10 5 460 466 10.2165/00019053-199610050-00003 10172868

[B32] Liljas B How to calculate indirect costs in economic evaluations Pharmacoeconomics 1998 13 1 1 7 10.2165/00019053-199813010-00001 10175982

[B33] Koopmanschap MA Rutten FF Van Ineveld BM van Roijen L The friction cost method for measuring indirect costs of disease Journal of Health Economics 1995 14 171 189 10.1016/0167-6296(94)00044-5 10154656

[B34] Shaw WD Feather P Possibilities for Including the Opportunity Cost of Time in Recreation Demand Systems Land Economics 1999 75 4 592 602 10.2307/3147068

[B35] Australian Bureau of Statistics Australian Bureau of Statistics. Canberra National Health Survey - Confidentialised Unit Record Files 2005

[B36] AIHW Australian Institute of Health and Welfare. Canberra Health system expenditure on disease and injury in Australia, 2000-01 Volume AIHW cat. no. HWE 26 2004

[B37] Australian Bureau of Statistics Australian Bureau of Statistics. Canberra How Australians Use their Time, 2006 Volume Cat no 4153.0 2008

[B38] Australian Bureau of Statistics Australian Bureau of Statistics. Canberra Labour Force, Australia, May 2007 Volume Cat no 6202.0 2007

[B39] Australian Bureau of Statistics Australian Bureau of Statistics. Canberra Average Weekly Earnings, May 2008 Volume Cat no 6302.0 2008

[B40] Magnus A Cadilhac D Sheppard L Cumming T Pearce D Carter R Economic Benefits of Achieving Realistic Smoking Cessation Targets in Australia Am J Public Health 2011 101 2 321 327 10.2105/AJPH.2009.191056 21164092 PMC3020195

[B41] Cadilhac DA Magnus A Sheppard L Cumming TB Pearce D Carter R The societal benefits of reducing six behavioural risk factors: an economic modelling study from Australia BMC Public Health 2011 11 483 10.1186/1471-2458-11-483 21689461 PMC3146859

[B42] Purchasing Power Parities (PPP) http://www.oecd.org/department/0,3355,en_2649_34357_1_1_1_1_1,00.html

[B43] Drummond MF McGuire A Economic Evaluation in Health Care: Merging Theory with Practice 2001 Oxford: Oxford University Press

